# The influence of lateral spinal curvature on range of motion

**DOI:** 10.1186/1748-7161-8-S1-P21

**Published:** 2013-06-03

**Authors:** N Nešic, V Šeper, E Davidovic-Cvetko

**Affiliations:** 1 University of Applied Sciences Lavoslav Ružicka Vukovar, Hungary

## Background

This study was conducted in Primary school on 97 pupils, age12±2, on 55 girls and 42 boys.

## Aim

The objective of this research was to establish the existence of lateral spinal curvature, as well as its influence on range of motion of the spine.

## Methods

Height, body mass, spacing between hands, and length of the upper limbs were measured in all of the examinees. Lateral spinal curvature was identified using a bob (plummet) and clinical examination. Examinees performed five spinal flexibility tests: right and left lateral mobility test, forward bending test, shoulder static flexibility test, and neck and trunk static flexibility test. Results gathered using a bob (plummet) and clinical examination were compared to the results of flexibility tests. Difference between these methods in children with, and without, lateral spinal curvature was determined with statistical T-test. Medcalc program was used for statistics. Statistical significance was affirmed at p=0.01 level.

## Results

Results on the prevalence of lateral spinal curvature in this study show less case in male population, 35.71%, over female population, 40%. Results of all four flexibility tests were in favor of healthy population. Neck and trunk static flexibility test showed difference of 4.11cm with statistical significance of p=0.0021, shoulder static flexibility test 4.41cm (p=0.0078), right lateral mobility test 2.62cm (p=0.0008), and left lateral mobility test 2.52cm (p=0.0017). There was no statistical significance for forward bending test.

## Conclusion

According to above mentioned results, it can be concluded that lateral spinal curvature has a considerable significance on spinal mobility.
